# Synthesis and Rheological Characterization of a Novel Salecan Hydrogel

**DOI:** 10.3390/pharmaceutics14071492

**Published:** 2022-07-18

**Authors:** Qinling Zhang, Teng Ren, Jing Gan, Lirong Sun, Chenxia Guan, Qian Zhang, Shihui Pan, Hao Chen

**Affiliations:** 1 Marine College, Shandong University (Weihai), No. 180 Wenhua West Road, Gao Strict, Weihai 264209, China; 202017683@mail.sdu.edu.cn (Q.Z.); sjtu.rt19960906@sjtu.edu.cn (T.R.); 202017661@mail.sdu.edu.cn (L.S.); guanchx@mail.sdu.edu.cn (C.G.); zhangqianzq@sdu.edu.cn (Q.Z.); panshihui@sdu.edu.cn (S.P.); 2 College of Life Sciences, Yantai University, No. 30 Qingquan Road, Laishan Strict, Yantai 264000, China; ganjing@ytu.edu.cn; 3 The Key Laboratory of Synthetic and Biological Colloids, Ministry of Education, Jiangnan University, No. 1800 Lihu Road, Wuxi 214122, China

**Keywords:** Salecan, hydrogel, thermal treatment, rheological

## Abstract

Salecan (Sal) is a novel microbial polysaccharide. In the present research, thermal treatment was performed to fabricate Sal hydrogel. The effect of Sal concentration on water holding capacity, swelling properties, texture properties, and microstructure of the hydrogels was discussed. It was found that the equilibrium degree of swelling (EDS) of Sal hydrogels was above 1500%, inferred Sal was a highly hydrophilic polysaccharide. As Sal concentration increased from 3.5 to 8.0 wt%, the hardness increased from 0.88 to 2.07 N and the water hold capability (WHC) increased from 91.3% to 98.2%. Furthermore, the internal network structure of Sal hydrogel also became denser and more uniform. Rheological studies suggested that elastic hydrogel formed under the gelation process. All these results demonstrated that Sal hydrogel prepared by thermal treatment had good gelling properties, which opened up a new safe way for the preparation of Sal hydrogel and broadened the application range of Sal.

## 1. Introduction

Hydrogels are polymeric network materials that can absorb and hold the bulk of water in their three-dimensional structures upon swelling [1]. The special three-dimensional network structure attracts considerable attention in various applications. Among the numerous polymers that fabricate hydrogels, polysaccharides have attracted huge attention due to their extraordinary properties such as safe, biodegradable, biocompatible, non-toxic, non-immunogenic, and comparatively cheaper, and suitable for human consumption [2]. Polysaccharide-based hydrogels have been applied in the fields of food industries [3], water treatment [4], drug carriers [5], wound dressings [6], tissue engineering [7].

Generally, polysaccharides can be classified into plant polysaccharides (such as pectin, cellulose, and carrageenan), animal polysaccharides (such as glycosaminoglycans, heparin and hyaluronic acid), and microbial polysaccharides (such as xanthan gum, cyclodextrin, and curdlan) according to their sources [8]. Based on the previous reports, microbial polysaccharides showed various bioactivities including anticancer [9], antioxidant [10], antimicrobial [10], cholesterol-lowering [11], prebiotic and immunomodulatory activities [12]. On the other hand, from the perspective of high production efficiency, short production cycle, and simple purification operation, microbial polysaccharides are more suitable for a wide range of applications in the food, pharmaceutical, and material industry [13,14].

Sal is a new type of anionic β-glucan harvested from the fermentation medium of the salt-tolerant strain Agrobacterium ZX09, which is composed of glucopyranosyl units connected by β-1-3-glycosidic bonds and α-1-3-glycosidic bonds [15]. As a new type of water-soluble polysaccharide, Sal has excellent rheological and nutritional properties and has great potential in the food industry and biomedical fields [15,16,17]. Studies have shown that Sal can be used as a dietary supplement, and the incorporation of Sal in the diet may improve gastrointestinal health [18]. Furthermore, Sal showed applicable properties as a stabilizer in improving the rheological performance and WHC of yogurt [19].

Strong hydrophilicity, good rheological properties, easy modification, biodegradability, and non-toxicity, all these properties make Sal an ideal material for forming hydrogels [20]. To date, the current research on Sal composite hydrogels has focused on using Sal as a filler in other networks, Sal does not form a hydrogel network [16]. Hydrogels with physically and chemically entrapped Sal can be fabricated by a variety of methods including free radical polymerization [21], cyclic freeze–thaw processing [22], graft copolymerization [23], and ionic crosslinking. Sal/PMA hydrogels were developed using a free radical polymerization approach for the controlled release of insulin. The release of insulin and the elasticity of the hydrogel can be adjusted by changing the content of Sal in the hydrogel composition [24]. Wei et al. reported that Salecan-g-PMAPTAC hydrogel fabricated by graft-polymerizing 3-(methacryloylamino) propyl-trimethylammonium chloride (MAPTAC) onto Sal chains. The swelling rate and mechanical properties of the Salecan-g-PMAPTAC hydrogel can be adjusted by the content of Sal [23]. The disadvantages of hydrogels prepared by free radical polymerization and graft copolymerization are that the process is complicated, and the initiators and crosslinking agents used have certainly toxic and harmful to some degree. Sal/PVA hydrogels prepared by repeated cyclic freeze–thaw processing. Sal can accelerate the degradation rate and markedly improve cell adhesion of the Sal/PVA hydrogel [22]. However, the disadvantage of the cyclic freeze–thaw method for preparing hydrogels is that the formed hydrogels usually have low swelling ratio and low transparency. Among the above hydrogels, Sal does not form a hydrogel network, so how to prepare the Sal hydrogel is still being researched. In addition to the above hydrogels, Sal-based metal hydrogels have been developed by Cr^3+^ [25]. However, Cr^3+^ is easily oxidized to Cr^6+^, which may cause carcinogenesis to human body. The above-mentioned methods have their own shortcomings, and finding a green method to prepare Sal based hydrogel so that it can be better applied in the food and biomedical fields is urgent.

In our preliminary experiment, ionic (Ca^2+^), freeze–thaw and thermal treatments were all adopted to induce Sal molecules to crosslink to form hydrogels, but only thermal treatment worked. To the best of our knowledge, this is the first study on the preparation of the Sal hydrogel. Swelling properties, texture properties, and microstructure of the hydrogel were evaluated to explore the optimum gelation condition. This report might open up a new way to prepare Sal hydrogel and broaden the application range of Sal in the biomedical industry.

## 2. Materials and Methods

### 2.1. Materials

Salecan (Mw~2000 kDa, 95% purity) was obtained from Sichuan Synlight Biotech Ltd. (Chengdu, China). NaCl (analytical grade) was purchased from Tianjin Guangfu Technology Development Co., Ltd. (Tianjin, China).

### 2.2. Preparation of Sal Hydrogels

The Sal hydrogels were prepared by heat treatment method. In brief, a certain amount of Sal powder was dissolved in 10 mL deionized water at room temperature and kept magnetically stirring for 3 h to prepare the Sal solution with a concentration of Sal (0.5, 2.0, 3.5, 5.0, 6.5, and 8.0 wt%). After keeping swelling overnight, the Sal solution was centrifuged at 4000 rad/min for 30 min to remove bubbles. Afterward, the Sal solution was heated at 50, 60, 70, 80, and 90 °C for 20 min, respectively, and then the heated samples were cooled to room temperature using running water to induce gelation. Subsequently, the hydrogels were stored in a refrigerator at 4 °C for 24 h to reinforce their mechanical strength.

### 2.3. Observations of Sol-Gel Transition

The Sal hydrogels with concentration of 2.0~8.0 wt% were prepared by the above method. Then, the hydrogel was reheated in a water bath at 90 °C for 20 min, and then cooled to room temperature again by running water, which was defined as a heating–cooling cycle. Finally, the state change of the hydrogel was observed during this cycle.

### 2.4. Characterization of Sal Hydrogel with Different Sal Concentration

#### 2.4.1. Appearance Observation of Sal Hydrogel

The prepared Sal hydrogels were placed on a prepared piece of paper marked with the letter “A” to evaluate the transparency of hydrogels [26].

#### 2.4.2. Measurement of Color Values

The color values of hydrogels with different Sal contents were measured by a precision colorimeter (NR110, Shenzhen Sanenchi technology co., Ltd., Shenzhen, China). The CIE Lab scale chromaticity parameters L*, a*, b* were recorded, respectively. L* denotes the brightness index, L* = 0 means black, L* = 100 means white; a* denotes red and green, +a* means red and −a* green; b* denotes blue and yellow, +b* is for yellow and −b* is for blue. The color values can objectively evaluate the magnitude of the chromatic aberration and its visual difference. The implications of general color values Δ*E* are shown in Table 1. Δ*E* can be calculated by the following equation [27].

(1) $$\Delta Ε=\sqrt{{\left(L^{*}-L_{c}\right)}^{2}+{\left(a^{*}-a_{c}\right)}^{2}+{\left(b^{*}-b_{c}\right)}^{2}}$$

where L*, a* and b* are the color parameter values of Sal hydrogels with a concentration of 5.0, 6.5 and 8.0 wt%, respectively. *L_c_*, *a_c_* and *b_c_* are the color parameter values of the control hydrogel (3.5 wt% Sal hydrogel).

#### 2.4.3. Textural Properties Analysis

The hydrogel samples were cut into cylinders (15 mm in diameter and 10 mm in height) and equilibrated (25 °C, 1 h) before performing textural measurements. Texture profile analysis (TPA) tests for tested Sal hydrogels were made at least in triplicate on a TMS-Pro (Virginia, VA, USA). Measurement methods were as follows: 60 mm/min compression speed, 30% compressive strain, and 0.3 N trigger force. Hardness, resilience, springiness, cohesiveness, gumminess, and chewiness were determined for each sample.

#### 2.4.4. Test on Water Holding Capacity (WHC), Water Content (WC)

WHC of the hydrogels was measured according to Bengoechea et al. [28] with some modification. Specifically, the hydrogel samples of approximately 5 g were weighed and then placed in ultrafiltration centrifuge tubes. Subsequently, the samples were centrifuged at 5000 r/min for 40 min at 4 °C, and then the water extracted by centrifugation was carefully absorbed with a filter paper. WHC of the hydrogels was calculated by weighing the samples before and after centrifugation. WHC was calculated with the following formula:

(2) $$\mathrm{WHC}\%=\frac{W_{2}}{W_{1}}\times 100$$

where *W*_1_ was the initial weight (g) and *W*_2_ was the final weight (g).

The WC was determined in a drying oven (DHG 101-00, Zhejiang, China) maintained at 80 °C (with air circulation) [27]. The hydrogels with a thickness of 3 mm were cut into squares of 2 × 2 cm, then dried in the oven at 80 °C for 4 h. The weights before and after drying were measured and the water content was calculated as follows:

(3) $$\mathrm{WC}\;\%=\frac{W_{o}-W_{d}}{W_{o}}\times 100$$

where *W_o_* is the initial weight (g) before drying, and *W_d_* is the final weight (g) after drying.

#### 2.4.5. Investigation of Swelling Behavior

The swelling behavior of these different hydrogels was investigated at room temperature. Briefly, the hydrogels were cut into cylindrical shaped specimens (3.3 cm in diameter) for drying in a vacuum freeze dryer. Lyophilized hydrogel samples were immersed in saline solution (0.9 wt% NaCl solution, pH = 6.3). At specific time interval (every hour), the hydrogels were taken out, gently blotted with filter paper to remove the surface water and weighed again until the weight of hydrogels reached constant value [29]. The swelling ratio (SR) and EDS of hydrogel was defined as follows [30]:

(4) $$\mathrm{SR}\%=\frac{W_{t}-wd}{W_{d}}\times 100$$

(5) $$\mathrm{EDS}\%=\frac{W_{e}-W_{d}}{W_{d}}\times 100$$

where *W_d_* is the weight of dried hydrogel, *W_t_* is the weight after swelling for predetermined time and *W_e_* is the weight of equilibrium swelling hydrogel.

#### 2.4.6. Rheological Analysis

Steady state rate sweep test: The steady-state shear rate scanning of Sal was carried out by the rheometer (Haake Mars 3, Thermofisher, Hennigsdorf, Germany) with a parallel plate geometry (35 mm in diameter, gap 1 mm). The relationship between Sal solution viscosity and shear rate was obtained to further determine the fluid type of Sal. Briefly, 2 mL of 1.0 wt% Sal solution was put into the rheometer and kept for 10 min, then pre-sheared for 3 min at a shear rate of 100 s^−1^ to obtain a homogeneous solution. Subsequently, the shear rate was increased from 0.001 s^−1^ to 1000 s^−1^, and the change of Sal solution viscosity was recorded.

Dynamic time sweep test: The gelation processes of Sal hydrogels with different concentrations were characterized by dynamic viscoelastic measurements with a strain of 5%. In brief, 2 mL of Sal solution with different concentrations was pipetted onto the plate of the instrument. Then, the edge of the geometry was covered with a layer of silicone oil to prevent water evaporation [31]. Time dependence of G′ and G″ for different samples was carried out at the frequency of 1 Hz. The scanning temperature increased from 25 to 80 °C at the speed of 5 °C/min, subsequently maintained at 80 °C for 20 min. Then, it was cooled from 80 °C to room temperature at the rate of −5 °C/min. The function of the storage modulus (G′) and loss modulus (G″) and the time was recorded during the gelation processes.

Dynamic frequency sweep test: The hydrogels obtained by the above dynamic time sweep were continued to carry on dynamic frequency sweep test. The test parameters were as follows: scan temperature was 25 °C, the strain was 5%, and the angular frequency was 0.1~10 Hz [32]. The function of the storage modulus (G′) and loss modulus (G″) and the angular frequency was recorded.

#### 2.4.7. Scanning Electron Microscope (SEM) Imaging

Field emission scanning electron microscope (Nova Nano SEM 450, FEI, Hillsboro, OR, USA) was employed to image the hydrogel samples. Prior to the test, all test samples were brittle-broken with liquid nitrogen to expose to fresh brittle sections. Subsequently, each hydrogel was freeze-dried to remove adsorbed water and coated with a thin layer of gold for 80 s to increase conductivity [33]. The SEM observations were performed at an accelerating voltage of 30 kV.

### 2.5. Statistical Analysis

The data were analyzed using SPSS Statistics 20.0 software and one-way analysis of variance (ANOVA) was used to determine the significant differences between each test (*p* < 0.05). All samples were performed in triplicate unless otherwise specified and all results were expressed as a mean ± standard deviation.

## 3. Results

### 3.1. Preparation of Sal Hydrogel

The effects of temperature and concentration on Sal hydrogels were summarized in Table 2. It can be seen that the gelation process of Sal is related to its concentration and heating temperature. When heating temperature was below 80 °C, Sal could not form the hydrogel. Meanwhile, when the concentration of Sal was less than 2.0 wt%, Sal hydrogel also could not be formed. However, as the concentration of Sal increased, the Sal hydrogels became more elastic and compact.

**Scheme 1:**
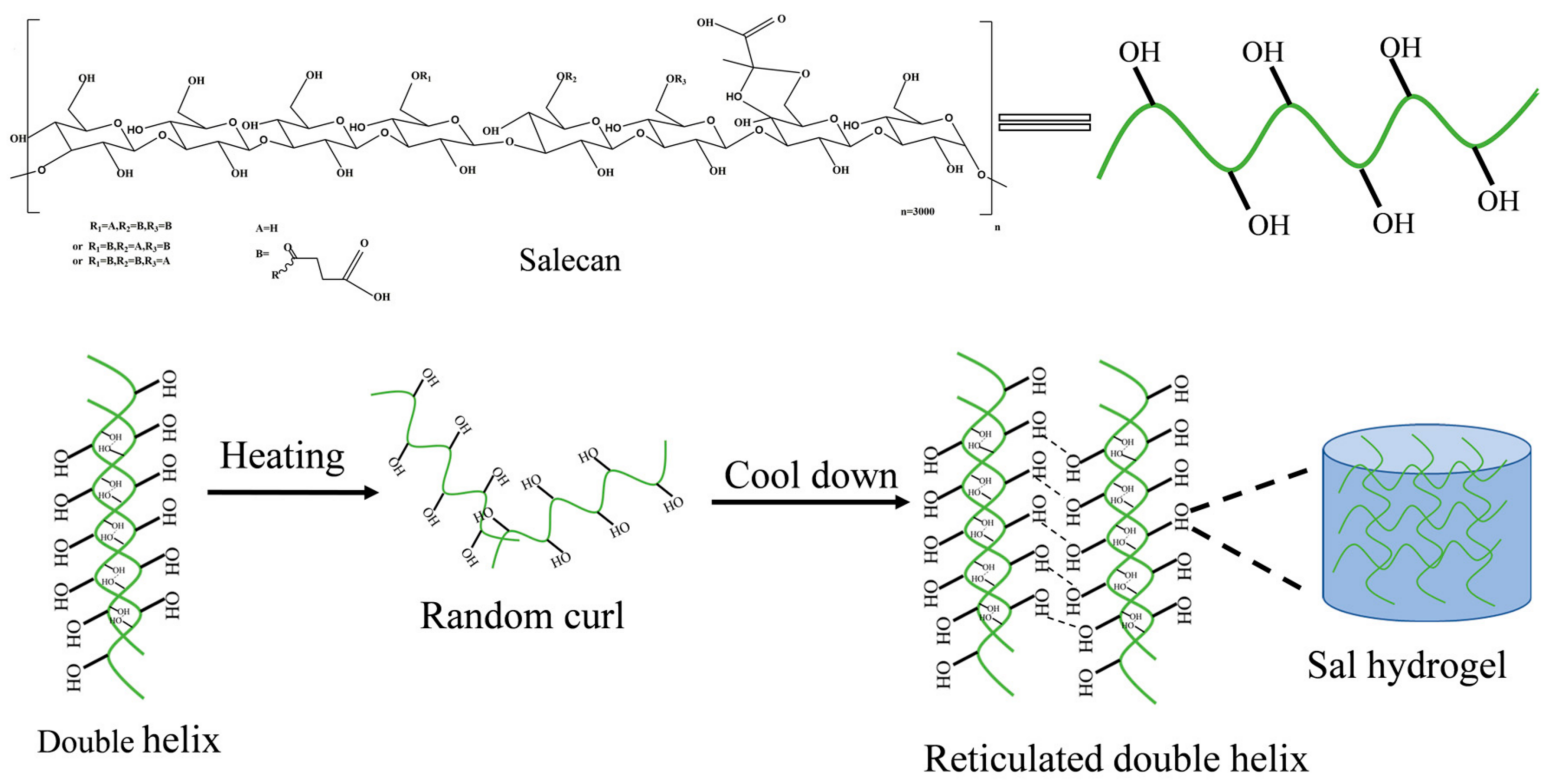
Preparation mechanism of Sal hydrogel.

The reason for this phenomenon may be determined by the molecular structure of Sal. When the concentrate ion of Sal was low, it was present as random coils in the solution. However, when the concentration was high, the Sal molecules would automatically aggregate to form a double helix structure with the help of hydrogen bonds, van der Waals forces and other forces. Additionally, at this time, the Sal solution system belongs to weak hydrogel system. The mechanism of preparing Sal hydrogel is shown in Scheme 1. Under heating conditions, the double helix structure of the Sal molecule unfolds and exists as a single strand, exposing more hydrophilic groups (-OH). When the temperature drops, the single-stranded molecules will automatically gather together with the help of -OH to reform a double helix structure that is more stable than before. Subsequently, the double helix can further aggregate to form a three-dimensional network structure through intermolecular hydrogen bonds, van der Waals forces, and other forces, which facilitate the retention of water molecules [34]. Finally, the Sal hydrogel was formed, thus realizing the transformation of the system from a weak hydrogel structure to an elastic hydrogel. The moldability of Sal hydrogel improves as the concentration of Sal increases, and the hydrogel structure tightens, which could be due to stronger interactions between molecules forming a more solid three-dimensional network structure [35].

### 3.2. Thermosensitivity of Sal Hydrogel

**Figure 1:**
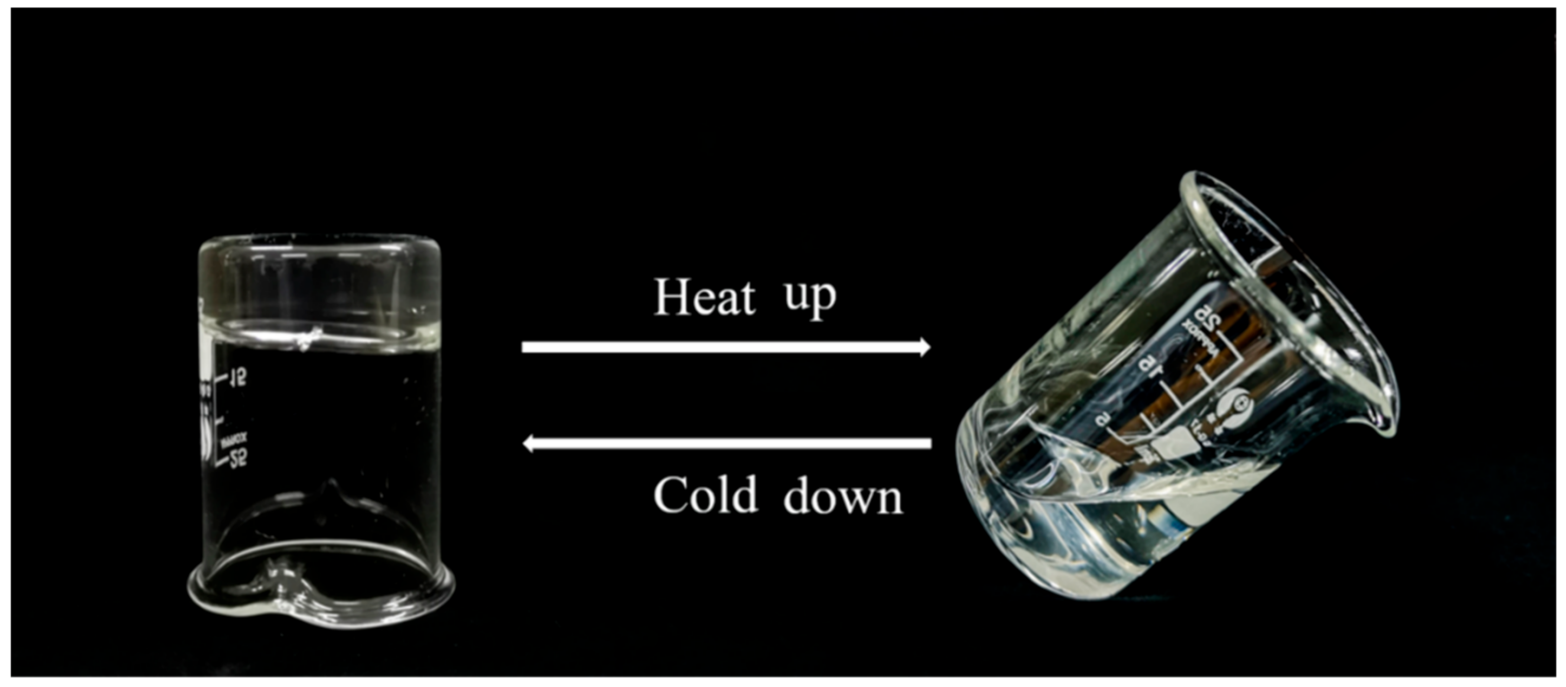
The thermally reversible conversion of Sal solution to hydrogel.

Thermosensitivity of Sal hydrogel was measured by the reheating method, and the results are shown in Figure 1 and Figure S1. The Sal hydrogels with a concentration of 2.0~8.0 wt% were reheated in a water bath at 90 °C for 20 min, and it was found that it could transform from the hydrogel state to the solution state. While cooling to room temperature, it turned into the hydrogel state again. This phenomenon shows that Sal hydrogel is a thermo-reversible hydrogel. Generally speaking, physical cross-linked hydrogels that form a cross-linked network structure by entangling polymer chains through non-covalent bonds are thermally reversible [36]. This phenomenon also proves that Sal hydrogel is a typical physical cross-linked hydrogel glue.

### 3.3. Characterization of Hydrogels

#### 3.3.1. Sensory Characteristics

**Figure 2:**
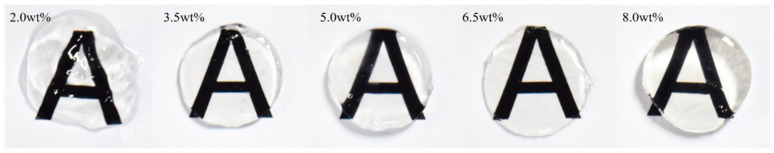
Images of the Sal hydrogel with different concentration.

The images of Sal hydrogels with different concentration are shown in Figure 2. With the increase in Sal concentration, the hydrogel appeared light yellow, but all samples were transparent. What is more, as the concentration of the hydrogels increased, the formability of the hydrogels improved. The formability of the 2.0 wt% Sal hydrogel was very poor, which had a rough surface and loose structure. While with the increase in the concentration, the surface becomes smoother and more compact. This might be attributed to the increase in concentration enabled by the contact of neighboring polymer chains, increasing the probability of intermolecular bonding, enhancing the interaction between Sal molecules while also improving the internal cross-linking of the hydrogel, resulting in stiffer hydrogels.

**Figure 3:**
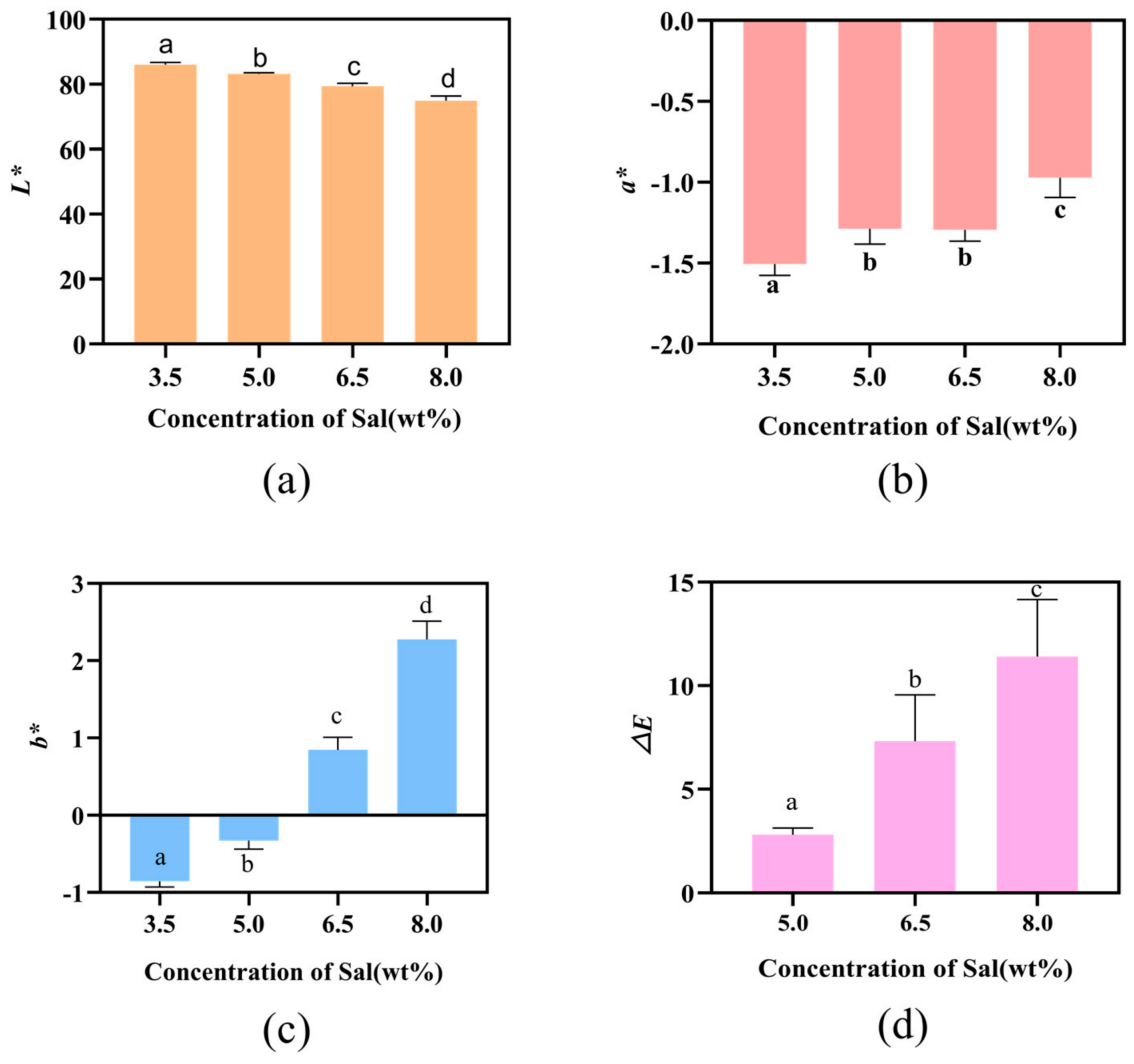
Changes in tested hydrogel colors: (**a**) L*, lightness/brightness; (**b**) a*, redness/greenness; (**c**) b*, yellowness/blueness; (**d**) Δ*Ε*, total color difference. Results are expressed as average, and different letters (a–d) indicate significant differences (*p* < 0.05).

The colors of the Sal hydrogels with different concentration are shown in Figure 3. Taking a Sal hydrogel with a concentration of 3.5 wt% as a control, the Δ*E* of the hydrogel of 5.0, 6.5, and 8.0 wt% are shown in Figure 3d. When Δ*E* was between 6.5 and 13, it meant the color difference between the test group and the control group was obvious. Therefore, when the Sal concentration was greater than 6.5 wt%, the hydrogels showed an obvious color change compared with 3.5 wt%. Figure 3c also further confirmed this phenomenon: since b* represented the blue-yellow value of the samples, +*b* was the yellow direction, and −*b* was the blue direction, when the Sal concentration increased to 6.5 wt%, the b* turned positive, which indicated that the hydrogel at this concentration started to appear light yellow, as the Sal concentration continued to increase, the b* value continued to increase, indicating that the concentration had a greater impact on the color of the Sal hydrogels. The reason for the pale-yellow color of the hydrogels with increasing concentration of the Sal might be due to the stronger interaction between molecular chains at higher concentrations, which promoted a denser network structure inside the hydrogel, thereby reducing the light transmittance of the hydrogel, showing the pale-yellow color.

#### 3.3.2. Textural Properties

Table 3 summarized the effects of Sal concentration on the texture properties of Sal hydrogel. As the concentration increased, the hardness, cohesiveness, springiness, and chewiness of the hydrogels also increased. Among them, the increase in the hardness could be related to the strength of the hydrogel, the increasing concentration promoted the interaction between the Sal molecules and facilitated the cross-linking of the hydrogel to form a denser network structure, thereby increasing its hydrogel strength. Springiness is an indicator of measuring the degree of hydrogel structure being undermined by the initial compression [37]. As the springiness decreases, the structure may break down into a few large pieces during the first compression, which results in hardly chewing, this is the reason for the increase in chewiness. Cohesiveness determines the strength of the internal bonds [33]. The concentration increases, and the cohesiveness is also elevated, indicating that the hydrogel structure is not easily destroyed, thus reflecting the denser network structure of the hydrogel [38]. In summary, as the concentration of Sal increased, the texture properties of the Sal hydrogel were better, which can better meet the needs of food processing and biomedical industry.

#### 3.3.3. Water Holding Capacity and Water Content

**Figure 4:**
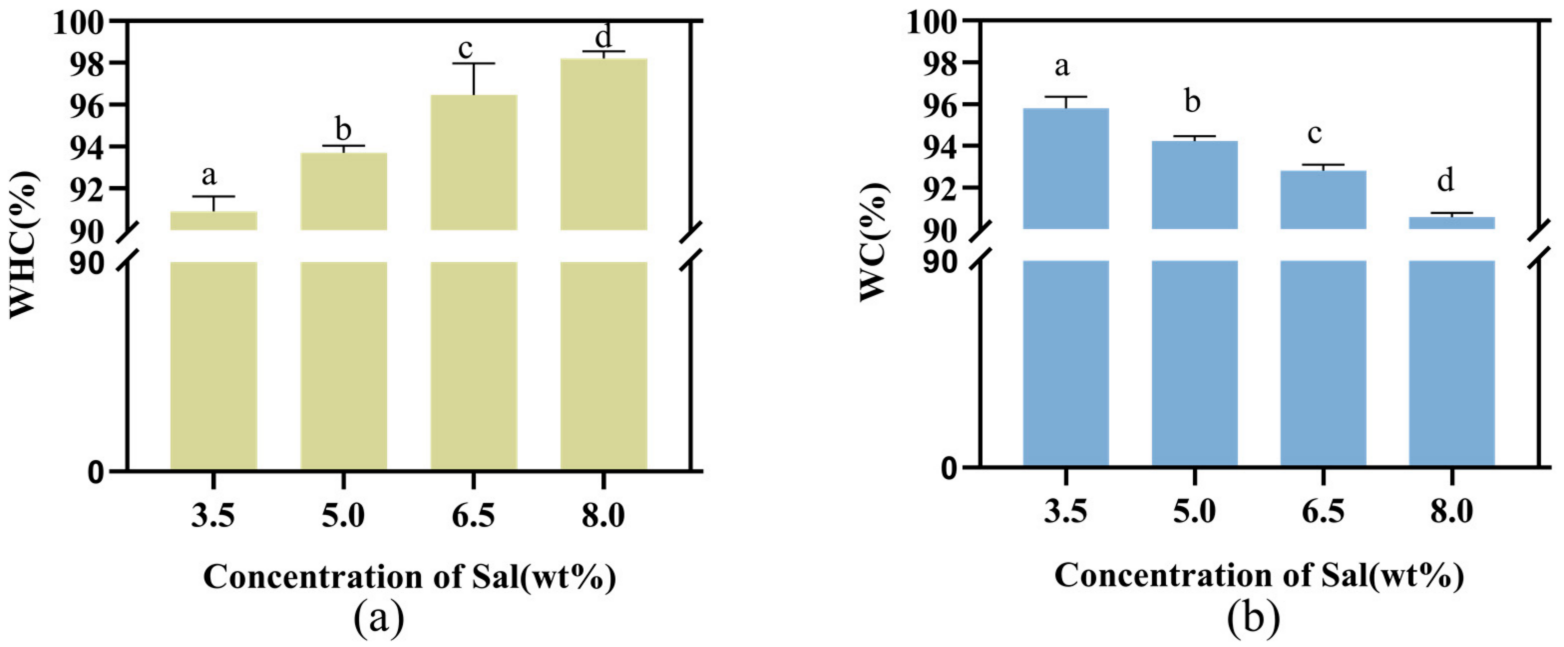
(**a**) The effect of Sal concentration on WHC of the Sal hydrogel. (**b**) The effect of Sal concentration on WC of the Sal hydrogel. Results are expressed as average, and different letters (a–d) indicate significant differences (*p* < 0.05).

As shown in Figure 4a, as the concentration increases, the WHC of the Sal hydrogel increased from 90.4% to 98.2%. This phenomenon might be due to the fact that WHC of hydrogels was dominantly affected by their internal microstructure and hardness [26,39]. Combined with the SEM results, with the increase in Sal concentration, the microstructure of Sal hydrogel became denser and more uniform. Such high WHC observed in this work was likely attributed to the uniform and dense crosslinked hydrogel network structure achieved from Sal after heat treatment. It has been proposed that a hydrogel with an evenly distributed network structure has higher WHC because water is more tightly trapped in the dense structure. The hydrogel network structure distribution becomes denser and more uniform as the concentration of Sal increases, and the water is more closely captured in the dense structure. In addition, the WHC is positively related to hardness [40]. Our findings reveal that WHC improved significantly by increasing Sal concentration, which had the same change trend with hardness.

The WC of the Sal hydrogels with different concentrations are presented in Figure 4b. As the concentration of Sal increased, the WC of the hydrogel dropped from 95.7% to 91.2%. However, the overall WC of the hydrogel was maintained above 90%, indicating that Sal had strong hydrophilicity, which might be attributed to the molecular structure of the Sal. A large number of hydroxyl groups are present on the Sal molecular chain, as one of the highly hydrophilic functional groups, it could promote Sal to absorb a large amount of water. As the concentration of Sal increases, the hydrogel network becomes tighter, resulting in a smaller hydrodynamic free volume, less water absorption, and ultimately a decrease in the water content of the hydrogel [41].

#### 3.3.4. Swelling Analysis

**Figure 5:**
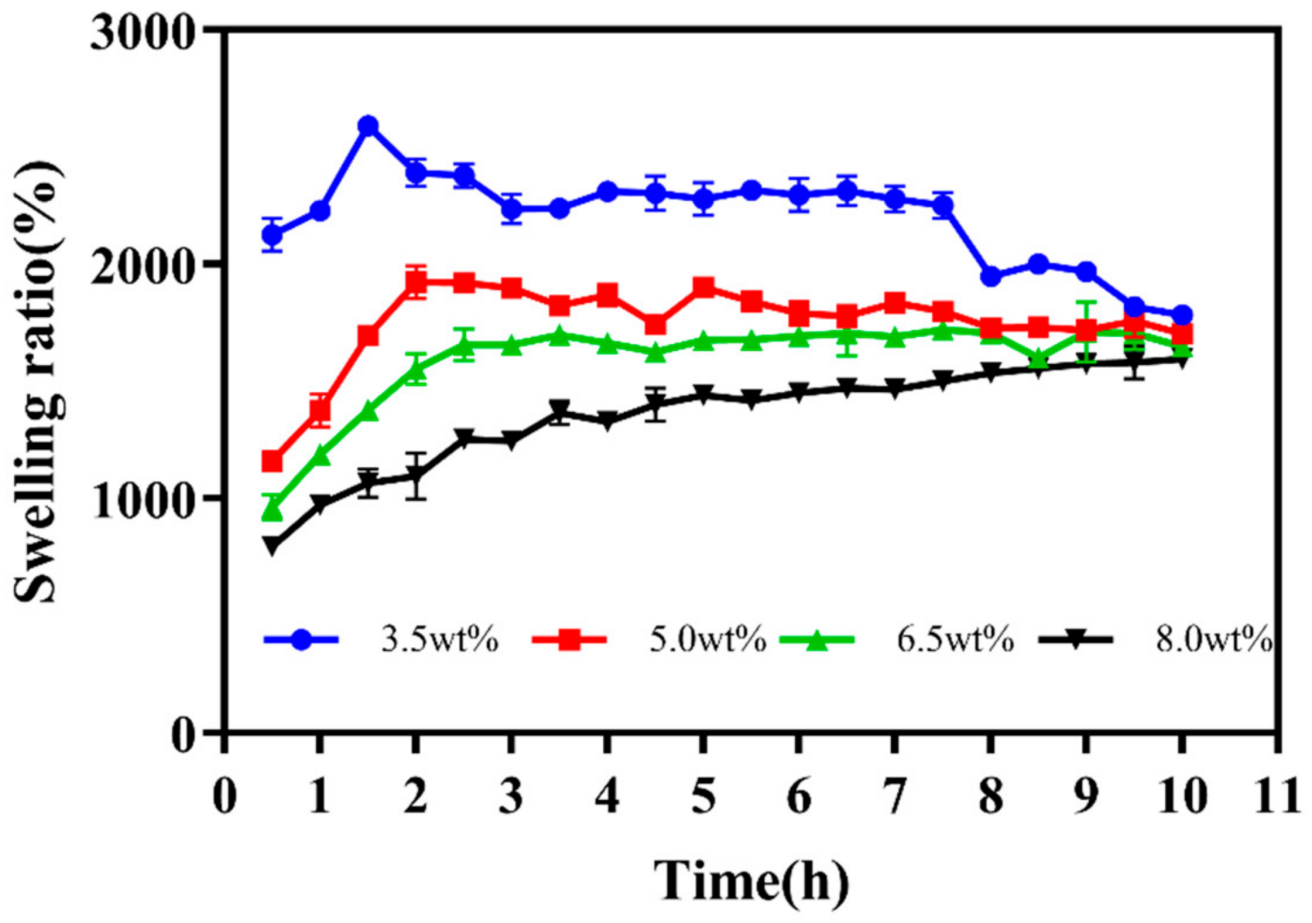
Swelling degree of Sal hydrogels with different concentrations.

The swelling ratio of Sal hydrogels with different concentrations in 10 h is shown in Figure 5. With the increase in swelling time, the swelling ratio of all the hydrogel samples increased first and then tended to be stable. Specifically, all samples showed a faster swelling ratio in the first 2 h, indicating that all these hydrogels had the ability to swell quickly. As the concentration increased, the longer it took for the hydrogel to reach equilibrium swelling ratio, which is attributed to the relationship between the internal structure of the hydrogel and its concentration: the greater the concentration, the denser the cross-linking of the hydrogel network, the longer it took to absorb water.

**Figure 6:**
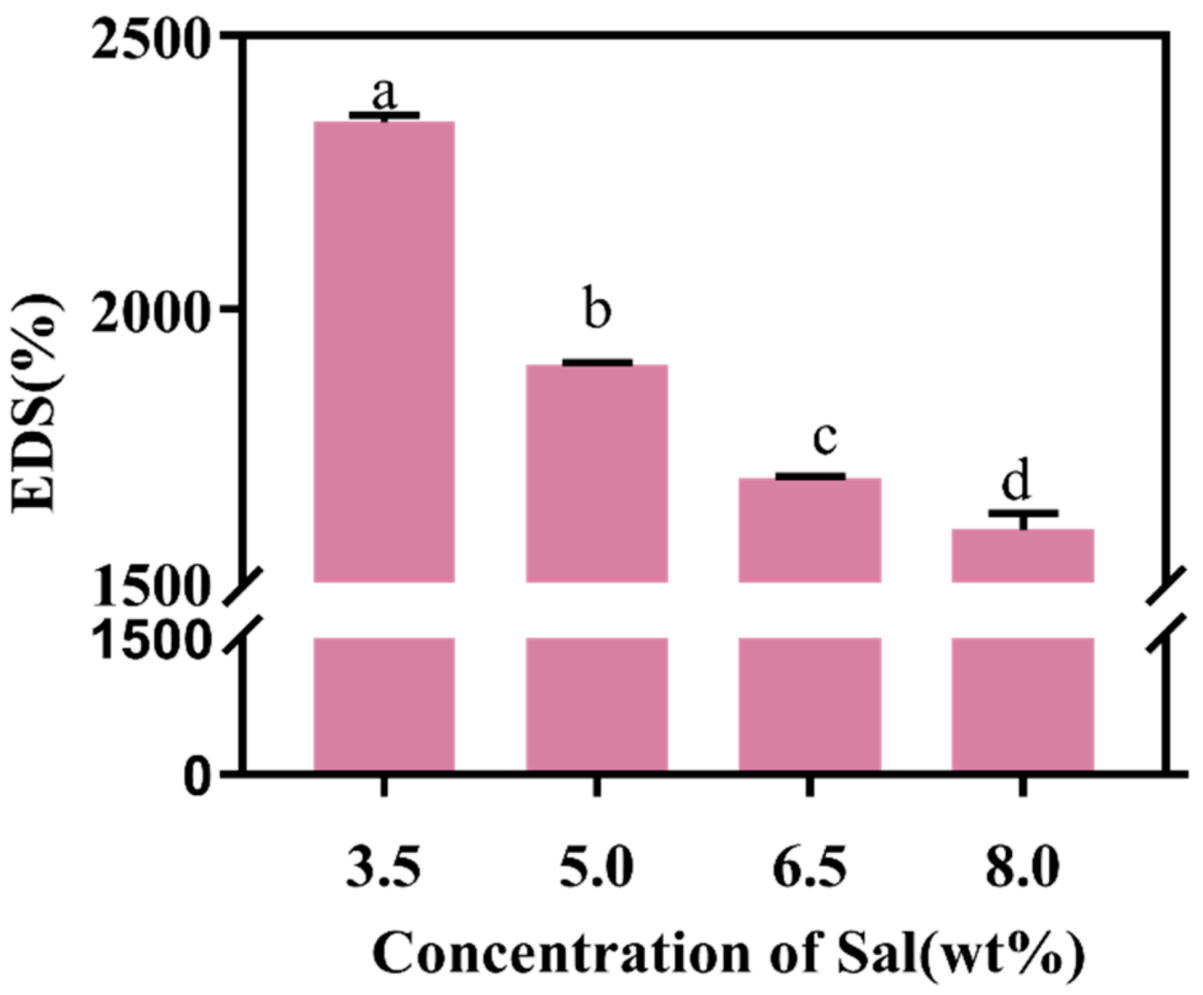
The effect of concentration on the EDS of Sal hydrogels. Results are expressed as average, and different letters (a–d) indicate significant differences (*p* < 0.05).

Figure 6 showed the effects of Sal concentration on the EDS of Sal hydrogels. All hydrogel samples had an EDS greater than 1500%, which was higher than most single polysaccharide hydrogels, indicating that Sal was a hydrophilic polysaccharide. As the concentration of Sal increased, the EDS of the hydrogels showed a downward trend. The rationale for this result could be that when the concentration of Sal was high, the molecular chain of polysaccharides was closely crosslinked, resulting in a denser network structure and smaller pore size. Therefore, its ability to absorb water has decreased, causing it to swell [42]. In addition, as the concentration of Sal increased, the hydrogen bonding force between the polysaccharide molecular chains was enhanced, which led fewer free hydroxyl groups remaining to absorb water [43]. As a result of these forces working together, the EDS of Sal hydrogel decreased as the concentration increased.

#### 3.3.5. Rheological Properties

**Figure 7:**
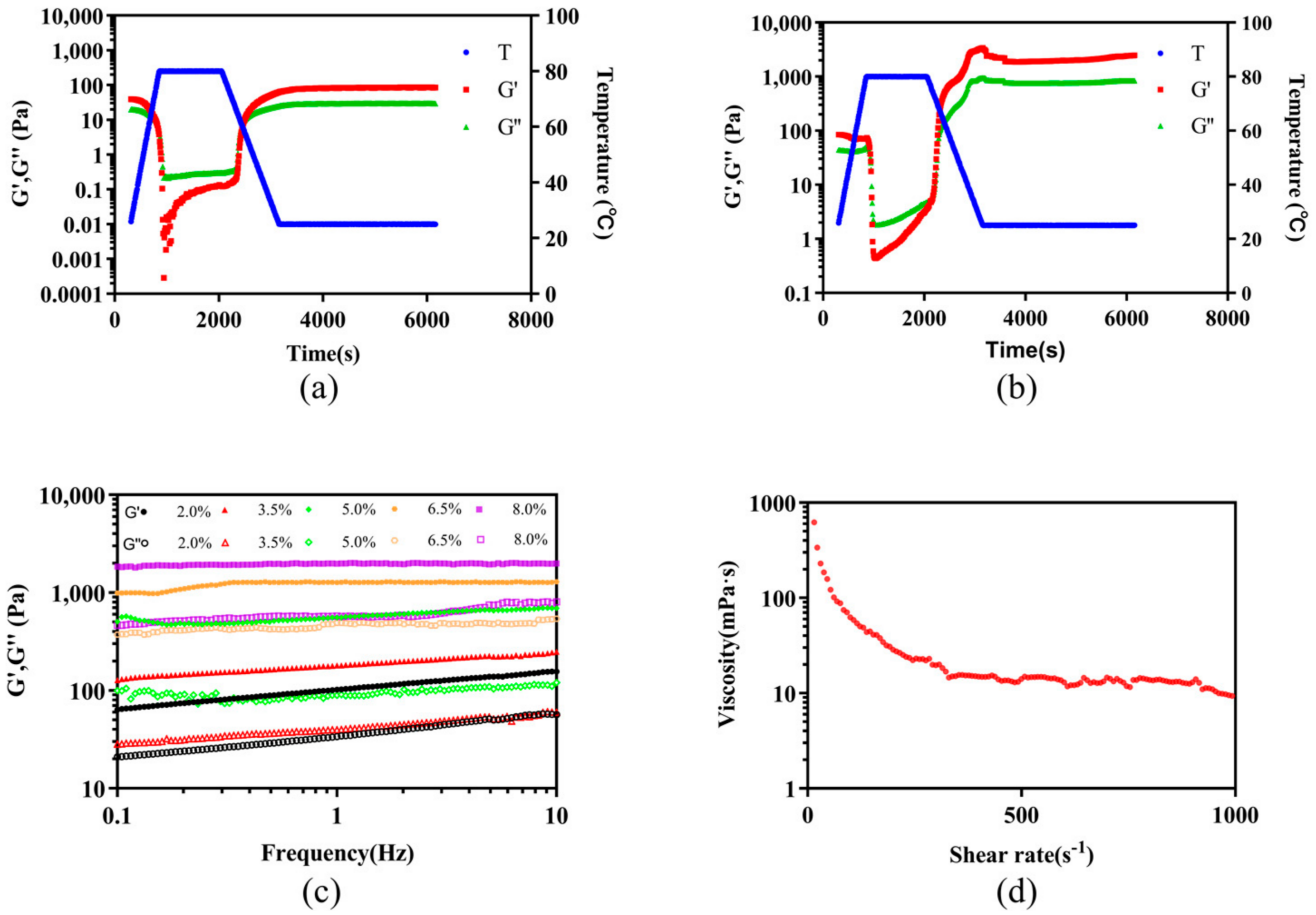
(**a**) Dynamic time sweep of 2.0 wt% Sal hydrogel. (**b**) Dynamic time sweep of 8.0 wt% Sal hydrogel. (**c**) Dynamic frequency sweep of all hydrogels. (**d**) Steady state rate sweep.

The time dependence of the storage modulus (G′) and the loss modulus (G″) for 2.0 and 8.0 wt% Sal solution at a constant frequency of 1 Hz are shown in Figure 7a,b. Obviously, at the beginning of the test, the G′ of these two Sal solutions were both greater than the G″, but the gap between G′ and G″ was small, showing obvious weak hydrogel properties. This may be attributable to the entanglement of the Sal molecular chain, which made the Sal solution exist as part of the double helical structure of the molecular chain to make the system exhibit a weak hydrogel state. However, both G′ and G″ of Sal showed a decrease during the heating process. This phenomenon could be attributed to the interaction forces such as hydrogen bonds between molecules that were destroyed at high temperature, resulting in the intensity of molecular activities exceeding the interaction forces between molecules so that the spiral chain began to untwist and became a pliable single chain [44]. While the temperature of the system was maintained at 80 °C, the G′ was less than the G″, indicating that the Sal spiral chain was completely untangled into a disordered chain, and the system appeared to be liquid-like. Moreover, both G′ and G″ increased sharply with time during the temperature drop to room temperature. Meanwhile, the intersection of G′ and G″ was observed, which corresponded to the gelation point [45]. In addition, the difference between G′ and G″ of the Sal hydrogel with a concentration of 8.0 wt% was significantly higher than that of a concentration of 2.0 wt%, which meant that the hydrogel had a good solid-like behavior when the Sal concentration was higher.

For all Sal hydrogels, the frequency sweep profiles of the G′ and G″ were tested. As can be observed from Figure 7c, Sal could still form a hydrogel at lower concentrations (2.0~3.5 wt%), but their G′ and G″ were highly frequency-dependent, indicating that a typical hydrogel was not formed. Nevertheless, when Sal was at a high concentration (5.0~8.0 wt%), G′ was nearly frequency-independent and much higher than G″ throughout the entire frequency range, indicating a gel-like material response [46], which also might be attributed to the fact that internal cross-linking of the hydrogel was denser at high Sal concentration.

The effects of shear rate on the viscosity of the Sal solution are shown in Figure 7d. Like most polysaccharides, the viscosity of the Sal solution decreased with the increase in the shear rate, indicating the pseudoplastic behavior. However, at the very low shear rate, we could not observe the Newtonian platform (viscosity does not change with shear rate) of the Sal solution, which might be caused by the following reason: there are many internal entanglement points in the system of long molecular chains, and it had the ability of untangling between molecular chains to form new entanglements, which could occur at a very low shear rate, so the Newton platform was difficult to recognize with the instrument.

#### 3.3.6. SEM Analysis

**Figure 8:**
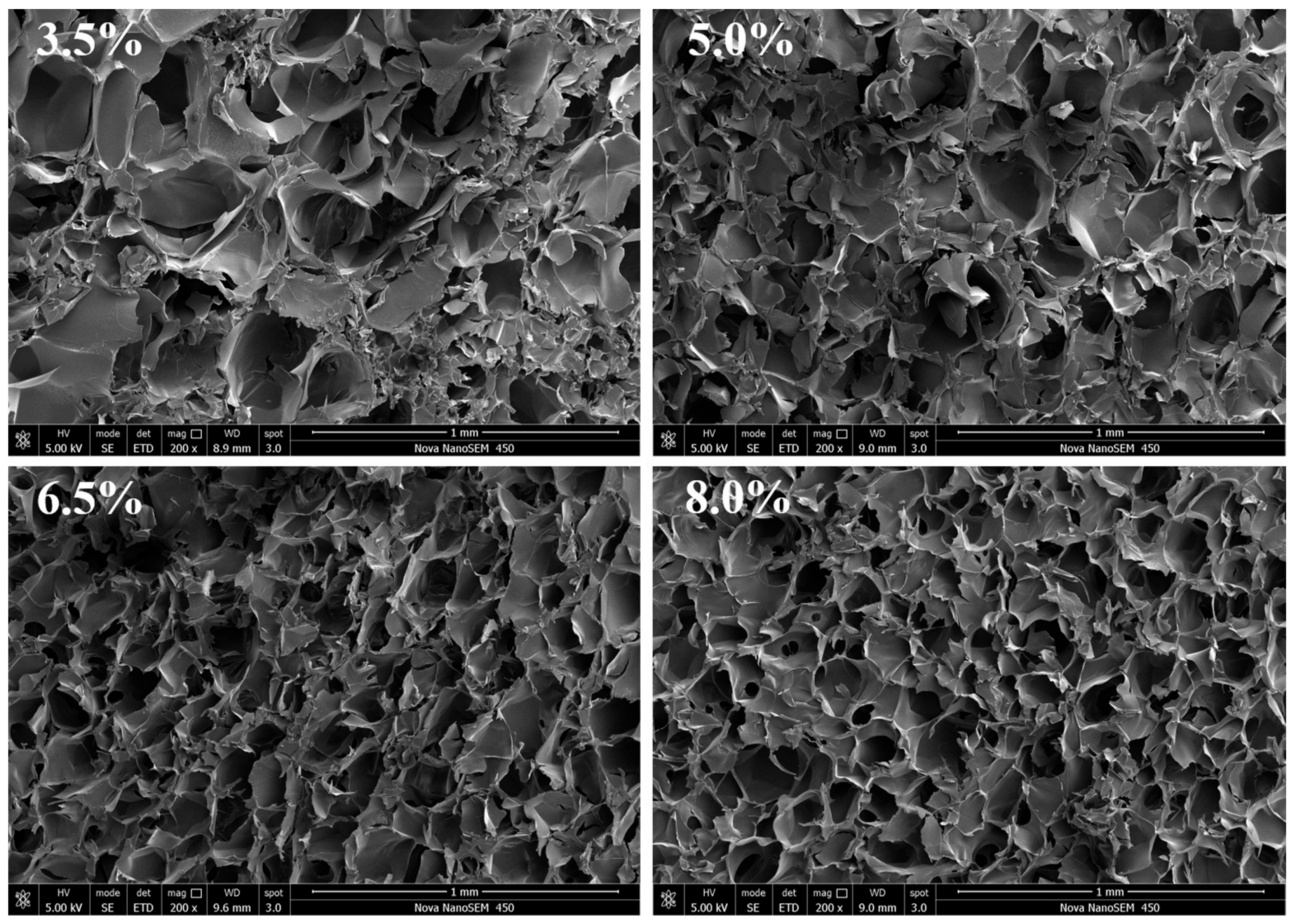
SEM micrographs of Sal hydrogels with different concentrations.

As a typical nutrition/drugs delivery carrier, the microstructure of hydrogels was one of the most important factors to be considered in selecting materials for delivery [47]. The cross-section morphologies of freeze-dried Sal hydrogels with different concentrations are displayed in Figure 8. With the increase in the Sal concentration, the microstructure of the Sal hydrogel becomes denser and more uniform. Meanwhile, the pore size gradually became smaller. These results were mainly related to the fact that as the concentration of Sal increased, it was conducive to the contact of adjacent polymer chains, thereby enhancing the molecular entanglement between polysaccharide chains, which caused a stronger molecular force and tighter network of the hydrogels, thus forming a hydrogel with a more stable structure [35].

## 4. Conclusions

In this study, the novel Sal hydrogel has been successfully fabricated by the thermal treatment method for the first time, which was a thermo-reversible hydrogel with good transparency. The formability, WC, WHC, swelling properties, texture properties, and microstructure of the hydrogel were markedly affected by the Sal concentration. As the Sal concentration increased from 3.5 to 8.0 wt%, the hydrogel hardness increased from 0.88 to 2.07 N, and the water hold capability increased from 91.3% to 98.2%. What is more, the internal network structure of Sal hydrogel also became denser and more uniform. In addition, shear rate scan confirmed that Sal solution was a pseudoplastic fluid without a Newtonian platform, and from the rheological properties of the hydrogel, we found that when the concentration of Sal exceeds 5.0 wt%, an elastic hydrogel could be formed. On the one hand, this approach could supply a safe way for developing the Sal hydrogel system. On the other hand, the thermal treatment method was suitable for the food and biomedical industry to broaden the application range of Sal.

## Figures and Tables

**Table 1:** General color values and their corresponding implications.

Color Values	Implications
1.6 < Δ*E* < 3.2	Cannot distinguish its color difference
3.2 < Δ*E* < 6.5	A few people can tell the difference in colors
6.5 < Δ*E* < 13	The color difference is very obvious
13 < Δ*E* < 25	Most belong to different colors
Δ*E* > 25	Different colors

**Table 2:** The effects of temperature and concentration on the Sal hydrogel formation.

	C (wt%)	0.5	2.0	3.5	5.0	6.5	8.0
T (°C)							
50		-	-	-	-	-	-
60		-	-	-	-	-	-
70		-	-	-	-	-	-
80		-	-	+	++	++	+++
90		-	+	++	++	+++	+++

Notes: “-” means the sample does not form hydrogel; “+” for the sample to form hydrogels which has poor formability and not compact enough; “++” for the hydrogels which has the compact surface; and “+++” for the hydrogels with better formability and more compact surface.

**Table 3:** The effects of Sal concentration on texture properties of Sal hydrogel.

Concentration (wt%)	Hardness (N)	Springiness (mm)	Chewiness (mJ)	Cohesiveness (%)
3.5	0.88 ± 0.12 ^c^	0.29 ± 0.66 ^b^	0.12 ± 0.06 ^b^	0.5 ± 0.01 ^b^
5.0	1.56 ± 0.04 ^b^	0.94 ± 0.04 ^a^	1.27 ± 0.12 ^a^	0.63 ± 0.06 ^a^
6.5	1.72 ± 0.09 ^ab^	1.18 ± 0.18 ^a^	1.47 ± 0.50 ^a^	0.63 ± 0.06 ^a^
8.0	2.07 ± 0.22 ^a^	1.21 ± 0.11 ^a^	1.73 ± 0.31 ^a^	0.70 ± 0.01 ^a^

Results are expressed as average, and different letters ^(a–c)^ indicate significant differences (*p* < 0.05).

## Data Availability

The data are available from the corresponding author.

## Supplementary Materials

The following supporting information can be downloaded at: https://www.mdpi.com/article/10.3390/pharmaceutics14071492/s1, Figure S1: The thermally reversible conversion of Sal solution to hydrogel: (a) 3.5 wt% Sal hydrogel. (b) 5.0 wt% Sal hydrogel. (c) 6.5 wt% Sal hydrogel. (d) 8.0 wt% Sal hydrogel.
