# Supplementary material for: Synthesis and Rheological Characterization of a Novel Salecan Hydrogel
Source: Pharmaceutics. 2022 Jul 18;14(7):1492. doi: 10.3390/pharmaceutics14071492 (PMC9323046; doi:10.3390/pharmaceutics14071492)
Supplement: Supplementary file 1 [file pharmaceutics-14-01492-s001.zip › pharmaceutics-1802229-supplementary.pdf]

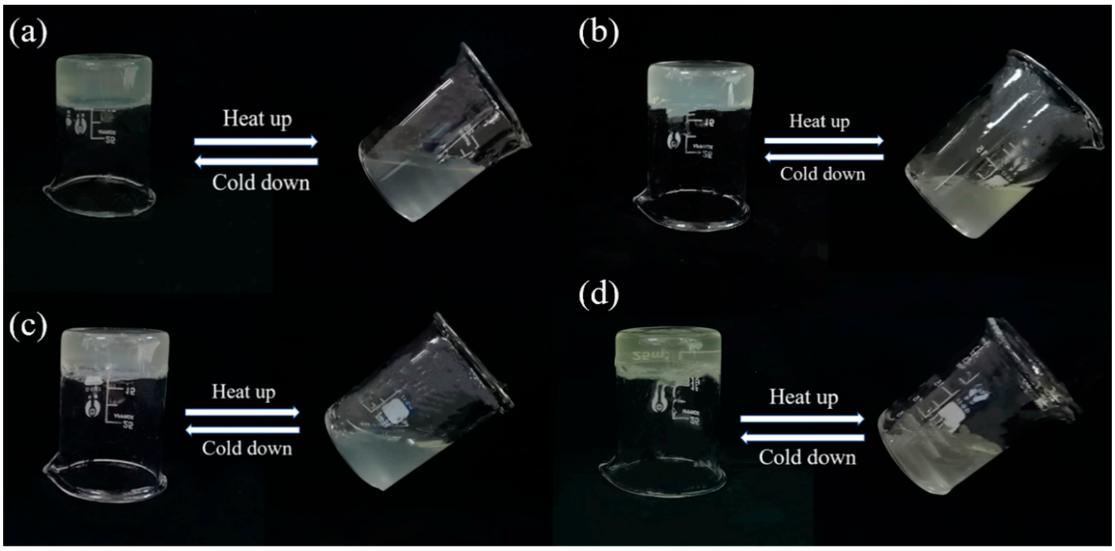

Figure S1. The thermally reversible conversion of Sal solution to hydrogel: (a) 3.5 wt% Sal hydrogel. (b) 5.0 wt% Sal hydrogel. (c) 6.5 wt%Sal hydrogel. (d) 8.0 wt% Sal hydrogel.
